# Survival and safety evaluation of *Bifidobacterium longum* subsp*. longum* ZS-8 in healthy adults, determined using PMAxx-qPCR and amplicon sequencing

**DOI:** 10.1128/spectrum.02861-24

**Published:** 2025-09-22

**Authors:** Feng Liu, Biao Dong, Zhongsun Wang, Dan Lin, Huan Xu, Baisheng Ke, Wanying Kang, Yang Jin, Xiuting Huang, Hui Lu, Liqing Zhao, Yun Qian, Liangling Cai, Long Xu, Zhenjiang Zech Xu

**Affiliations:** 1 State Key Laboratory of Food Science and Technology, Nanchang University 547468, Nanchang, People's Republic of China; 2 Shenzhen Wanhe Pharmaceutical Co., Ltd., Shenzhen, People's Republic of China; 3 Department of Gastroenterology and Hepatology, Shenzhen University General Hospital 558113 , Shenzhen, People's Republic of China; 4 College of Bioscience and Bioengineering, Jiangxi Agricultural University 91595, Nanchang, People's Republic of China; 5 Department of Food Science and Engineering, College of Chemistry and Environmental Engineering, Shenzhen University 618851 , Shenzhen, People's Republic of China; 6 Marshall Laboratory of Biomedical Engineering, Shenzhen University 47890, Shenzhen, People's Republic of China; 7 Department of Geriatrics, Huazhong University of Science and Technology Union Shenzhen Hospital 194030, Shenzhen, People's Republic of China; Shandong University, Jinan, China

**Keywords:** probiotics, PMAxx, live/dead bacteria, gut microbiota

## Abstract

Species-level quantitative PCR (qPCR) provides in-depth knowledge of oral probiotics in the human gastrointestinal tract (GIT). However, it lacks the capability to differentiate exogenous strains from native microbiota, nor can it distinguish between live and dead bacteria. In this study, we employed improved propidium monoazide (PMAxx)-qPCR to evaluate the survival and colonization of *Bifidobacterium longum* subsp*. longum* ZS-8 (designated ZS-8) on the strain level in the GIT and its impact on human gut microbiota. By spiking in live and dead ZS-8, we demonstrated that strain-level PMAxx-qPCR could identify and quantify the viable ZS-8 in fecal samples accurately. Using this method, we found that, in healthy humans, oral administration of ZS-8 can transiently survive in the GIT, and multi-layer seamless capsules (MLSC) significantly improve the gastrointestinal tolerance and survivability of ZS-8 compared to its powder form. Furthermore, through selective cultivation and PMAxx-microbiome sequencing, we investigated the response of gut viable microbiome to ZS-8. Results showed that, while the microbiota diversity and total viable counts of *Bifidobacterium* and *Lactobacillus* remained stable, certain indigenous species of *Bifidobacterium* and *Lactobacillus* increased in abundance, confirming ZS-8's probiotic potential in healthy individuals. Overall, our study demonstrates the effectiveness of combining strain-specific comparative genomics with PMAxx-qPCR for evaluating probiotic survival and colonization in the human gut and highlights the safety of ZS-8 oral administration in healthy individuals.

**IMPORTANCE:**

The survival and colonization of probiotics in the gut are critical for their functional efficacy, yet conventional species-level quantitative PCR (qPCR) fails to distinguish exogenous strains from native microbiota or differentiate live from dead bacteria. By integrating strain-specific comparative genomics with propidium monoazide (PMAxx)-qPCR, we precisely quantified the viability of *Bifidobacterium longum* ZS-8 at the strain level in the human gut after its oral administration. Our study demonstrated that 1.53–6.90% of cells surviving transit and multi-layer seamless capsules (MLSC) significantly enhanced the gastrointestinal tolerance of ZS-8. While ZS-8 administration did not alter gut microbiota diversity or total viable counts of *Bifidobacterium* and *Lactobacillus*, it selectively increased the abundance of specific indigenous beneficial species. This method overcomes the dual limitations of traditional techniques (strain-level specificity and viability discrimination), providing a robust tool for probiotic research. Furthermore, our findings confirm the safety of ZS-8 in healthy individuals and its potential to modulate gut ecology, offering a scientific foundation for personalized probiotic development and clinical translation.

## INTRODUCTION

*Bifidobacterium* spp. are a crucial group of beneficial gut bacteria, well-supported by extensive clinical evidence (1). Recent research has strongly indicated that low levels of *Bifidobacterium* are associated with various adverse clinical conditions (2). Positive modulation of gut microbiota through external intervention with *Bifidobacterium* can have a profound impact on host health (3). Introducing *Bifidobacterium* strains to the human gut has been reported to ameliorate conditions such as constipation (4), antibiotic-associated diarrhea (AAD) (5), irritable bowel syndrome (IBS) (6), and inflammatory bowel disease (IBD) (7, 8). Additionally, improvements have been observed under allergic conditions, including allergic rhinitis (9) and atopic dermatitis (10, 11). This has led to an increased interest in the therapeutic use of the oral probiotics. *Bifidobacterium longum (B. longum*), which colonizes the human gut from infancy (12), is broadly distributed across individuals of various ages (13) and is recognized as a core member of the human microbiome (14). *B. longum* plays a vital role in protecting against pathogens, regulating the immune system, producing vitamins, and utilizing lactose (15), thereby promoting intestinal homeostasis and overall host health. ZS-8 was isolated from the feces of a healthy infant. It was screened from a library of lactic acid bacteria (*n* = 200) at Wanhe Pharmaceutical based on its unique properties. ZS-8 possesses an elongated cellular morphology, which facilitates high-density fermentation and cost-effective production. Additionally, ZS-8 demonstrates significant antimicrobial activity and robust acid production (Table S17).

Probiotics are defined as live microorganisms that, when ingested in adequate amounts, confer a health benefit on the host (16). To exert their effects, *Bifidobacterium* spp. must tolerate harsh environments and survive gastrointestinal transit (17). However, *B. longum* exhibits low tolerance to acid, bile salts, and digestive enzymes (18). Consequently, a significant proportion of these bacteria are exposed to such harsh conditions, resulting in structural damage and loss of biological activity, which diminishes their effective dose in the gastrointestinal system (19). Riaz et al. pointed out that free *Bifidobacterium bifidum* significantly decreased from 10.96 to 3.03 log CFU/g when exposed to simulated intestinal fluid (SIF) after 4 hours (20). Mao et al. also found that the viability of free *B. longum* cells was reduced by 5.58 log units when exposed to simulated gastric fluid (SGF) for 60 minutes (21). Various formulations have been reported to enhance the resistance of sensitive *Bifidobacterium* spp. against adverse environments (22). Encapsulation or microencapsulation is one of the most efficient methods (23). In this study, we employed a multi-layer seamless capsule (MLSC) technology to encapsulate the sensitive ZS-8 strain. The outer layer, composed of specialized proteins, provides structural hardness. The middle layer, formed by a hardened oil membrane, acts as a barrier against acid and oxygen, thereby effectively safeguarding the live bacteria in the innermost layer (23). Although *in vitro* studies have demonstrated that MLSC can enhance the survival rate of ZS-8 in SGF and SJF, there is no relevant research on whether MLSC can similarly increase its survival *in vivo*.

To accurately assess the survival rate of exogenous *Bifidobacterium* in the gut, it is crucial to establish a reliable method for strain-level identification and quantification. Current general approaches include plate-counting and species-level PCR (24). Although plate-counting can directly reflect viability, it is time-consuming, often inaccurate, and unable to distinguish between exogenous and endogenous strains of the same species. *B. longum* can be endogenously distributed in the gut of individuals of various ages (13). Due to the 100% sequence similarity of 16S rDNA within the same species of *Bifidobacterium*, species-level quantitative PCR (qPCR) cannot differentiate the target strain from phylogenetically related species present in the baseline microbiota (24). Moreover, DNA extraction does not distinguish between live and dead cells, making DNA-based PCR technology unable to differentiate viable microbes from dead ones (25). Consequently, these common methods are thus inadequate for accurately quantifying the viable target strain in the gut. Therefore, advanced strain-level identification and quantification methods that specifically target live *B. longum* are essential for accurately assessing its survival and colonization in the gut, as well as for understanding its functionality and mechanisms of action (24). Recently, with the growing availability of sequenced *B. longum* genomes, strain-specific gene markers have become accessible, enabling the identification of *B. longum* at the strain level (24). Furthermore, propidium monoazide (PMAxx), a photoreactive DNA-binding dye, has been shown to selectively bind to exposed double-stranded DNA, thereby preventing PCR amplification of DNA originating from dead cells (25).

Here, we first developed ZS-8 strain-specific primers by conducting a comparative genomics analysis involving 553 strains of *B. longum*. The viability of ZS-8 was subsequently quantified and validated in human fecal samples using PMAxx-qPCR technology. Following this, a 2 week intervention trial was conducted to investigate the gut microbiota's response to various preparations and doses of ZS-8 in healthy volunteers. This study aims to demonstrate the feasibility of combining strain-specific comparative genomics with PMAxx-qPCR for accurately analyzing the survival and colonization of probiotics in the human gut. Additionally, we explored the impact of ZS-8 on the composition and diversity of gut microbiota in healthy individuals, providing valuable insights into its safety and potential benefits for gut health.

## MATERIALS AND METHODS

### Bacterial strains and culture conditions

ZS-8 was obtained from Wanhe Pharmaceutical Co., Ltd. (Shenzhen, China). Additionally, 72 bacterial strains (14 strains of *Bifidobacterium* spp. and 58 strains of other bacteria) were acquired from HB-CICC (Wuhan, Hubei, China) (Table S1). The strains of *Bifidobacterium* spp. and *Lactobacillus* spp. were inoculated into MRS medium at 37°C for 1–2 days. Other anaerobic bacteria were cultured in WCA broth at 37°C for 1 or 2 days. All anaerobic bacteria were cultured under anaerobic conditions using a Bactron Z-1 chamber (Cornelius, USA). Aerobic bacteria were cultured in LB broth at 37°C with shaking at 180 rpm.

### Survival of free and encapsulated ZS-8 in SGF and SIF

The viability of ZS-8 was determined using an *in vitro* digestion model as described by Chinese Pharmacopoeia (2020). For the gastric fluid tolerance test, SGF was prepared by dissolving 5 g pepsin in 500 mL distilled water and pH was adjusted to 1.5 using HCl or NaOH. To assess tolerance to intestinal fluid, 5.0 g of trypsin and 1.5 g of bile salts were added to a sterile solution containing 3.4 g potassium dihydrogen phosphate, dissolved in 500 mL distilled water. The pH was adjusted to 6.8 with 0.5 M NaOH. Both SGF and SIF were sterilized by filtering through a 0.22 µM membrane.

Tolerance studies were conducted using a VK7000 dissolution test apparatus (Tianda Tianfa, Tianjin, China). Free cells and encapsulated cells, each weighing 0.5 g, were placed in separate sample baskets and dissolved in 500 mL sterile SGF for 2 hours. The samples were then transferred to SIF for an additional 4 hours, and both dissolution tests were performed under constant agitation (100 rpm) at 37°C. Viability was assessed by taking samples at 120 minutes (end of SGF exposure) and at 360 minutes (end of SIF exposure). The number of viable cells was quantified using plate-counting and expressed as log10 CFU/g.

### Bacterial genome sequencing and retrieval of publicly available genomes

The complete genome of ZS-8 was sequenced using the Illumina Novaseq PE150 platform. Sequencing libraries were prepared with the NEBNext Ultra DNA Library Prep Kit for Illumina (NEB, USA), following the manufacturer's recommendations. Index codes were added to attribute sequences to each sample. Briefly, the DNA sample was fragmented by sonication to a size of approximately 350 bp, then DNA fragments were end-polished, A-tailed, and ligated with the full-length adaptor for Illumina sequencing, followed by PCR amplification. Finally, PCR products were purified using the AMPure XP system. The libraries were analyzed for size distribution with an Agilent 2100 Bioanalyzer and quantified by real-time PCR. The sequencing was performed at Beijing Novogene Bioinformatics Technology Co., Ltd., resulting in 3 GB paired-end raw reads. Adapters and low-quality reads were removed from the raw data. The remaining clean reads were assembled using SPAdes software. Moreover, a total of 552 publicly available *B. longum* genomes were downloaded from the National Center for Biotechnology Information (NCBI) database for comparative genomic analysis.

### Design and validation of strain-specific primers

The 553 genome sequences (comprising 552 publicly available strains and the ZS-8 strain) were re-annotated using Prokka (26), and a pangenome analysis of the resulting protein sequences was performed with Roary, applying a minimum BLASTP percentage identity of 90% (27). Genes unique to ZS-8 and absent from the other 552 strains were initially identified. A nucleotide database containing 553 *B. longum* genomes (Table S9) was then conducted using the makeblastdb command, and the strain-specific gene sequences were analyzed via BLASTN against this database. DNA sequences that were only present in the ZS-8 strain were retained (Table 1). Subsequently, the specificity of these DNA sequences was assessed by performing a nucleotide BLAST against the NR/NT database of NCBI. Finally, DNA sequences exhibiting no hits in the database were selected.

**TABLE 1:** Strain-specific genes identified for ZS-8

Gene designation	Gene sequence
>HPHHGGJE_01475 hypothetical protein	ATGGGCGAGCATGATCCGCATAATCTGGCCTGCGTGCTCGTCGCGGACGCGTACTGGAATCTGGTGGGGCCGACGG CCGCGTTCGGCCGGCGCGACCGGCTGGACGCGGACCGCGTCCTCTCCTGGTATCGCGCGCACGCCGGCGAATTGA GACAGGCGACGAGGGACGGCCGTTACGTGGCCCTGCTCTCCGACGGGCGTCTTCCCGCGTCGGCGGCCGGCCCCG TGCTGGCGAACATCCGACGCGTCGCCCCGGACGCATGCGACCGGTTCCTGGGCCTGCTCGCCGACCCGGCGACCCC GGTGCGCCGCCGGTTGGACGAGGGTTTGGACCGGTCGGACGGGCGCGGCTCCATCCGTTTCAACGCGGGCCTGCTC GTCAAATGCTGGAACTTGACCGGACTCGGCGCATCGCTGCCGCTCCCCTTCTAG
>HPHHGGJE_01548 hypothetical protein	ATGAAACCGTTTGACGAACACTTCGAACACCGGCTGCTCGACCATTTCGCCTACGACACCCGCACCGACGACGGATACG GCCCCGACATCCGCGCGTCGGCGACCGTTGCGCCCGTTCGTTCCCCAACGTCGGACAACGAGTGTTCTTCCGCATCGA ACGCGAGCCGAATACCGGCCTGCCGTACTGTGTGTTCGACGACGGGTCTTGGAACAGTCTGCTATGCACGTTCCTCAAA AACGGCAAAGGATGCGCGATCGTCCTGCTTGGACGGCCGCACGGCATCGCAACGCCATGAGTCGATGGGCATACGATC GGACCCGTACGCACTCGACCCCGGCATTCGAGGGCGGGCCGGAGACGCGCTTCGGCCCGCGGCCGGAAAGGCCGGC CGAACGCCGTATACAGGCCGGTCTGCGACCCGGGTACCCATCCCCGCCCTCAACCCGTACGCGGGGAAGGAGGCGGACTGA
>HPHHGGJE_01549 hypothetical protein	ATGGAATGCGCGGTATGTTCCACGATGGCGGATCCCGAGTCCATGAACCTATGCGCATGCTGCGAAAGACCCGTCTGCGA CAACTGTGTTCATCTGAACGCATACGGCGAACCAATCTGCTTCCAATGCTGGGAAGGTGAATAG
>HPHHGGJE_01550 hypothetical protein	ATGTCGGCCACGCCGGCCGTCGGAGAACATGATGATTCAAGGTCGGTATCCGAGGCCGACTGGCTGGAATTCGATTCCGA GGCCAACGACTACGGGGTGCCGGACCGGGCCGGCCTGCAATCAGTCATCGACGCGATAGGCGGGCATTATCTGCATCTCG CCCGCGAATACGGGTGGCTCGACACCGAGGTGCGCGACGCGGTGTACGTGGAGACGAAACGGAGAGGATGGTGCGGGGAATGA

Based on the unique DNA sequence of ZS-8, we designed the qPCR primers with primer 5.0 software. Subsequently, the specificity of these DNA sequences was verified using Primer-BLAST on the NCBI website. We used qPCR of 39 bacteria to validate the specificity of primers. For validation of the intra-species and subspecies specificity, eight *B. longum* strains were used. For the intra-genus specificity, six different *Bifidobacterium* strains were selected. Additionally, 25 gut bacteria members and eight human fecal samples were included for specificity testing (Table S1).

### LyPMAxx treatment and DNA extraction

The lyPMAxx treatment of pure bacterial cultures or fecal samples was conducted as described in our previous study (25). The processed fecal suspension (500 µL) or ZS-8 (500  µL) was centrifuged at 14,000  rpm for 15  min at 0°C, after which the cell pellet was collected and resuspended in 500 µL of sterile water. Following a brief vortexing, the samples were allowed to stand at room temperature for 5  min. To each sample, 13.4  µL PMAxx (Biotium, CA, USA; 2 mM) was added and incubated in the dark for 10  min. The tubes were then positioned horizontally (<20  cm) under an LED blue light (470  nm; 60 W) for 15  min, with gentle manual inversion and shaking every 5  min. After light exposure, samples were stored at −80°C until DNA extraction.

For bacterial strain, DNA was extracted using a Takara bacteria genomic DNA extraction kit (Takara, Kyoto, Japan) in accordance with the manufacturer's instructions. For human fecal samples, DNA was extracted using the PowerFecal Pro DNA kit (QIAamp, MO BIO Laboratories, Carlsbad, CA, USA). DNA was eluted in 60 µL Qiagen elution buffer.

### Quantitative PCR assay

A quantitative PCR assay was conducted utilizing three pairs of specialized primers targeting distinct cells (Table 2): *Bifidobacterium* spp. (28), *Lactobacillus* spp. (29), and ZS-8. Additionally, a pair of bacterial universal primers targeting the V6 regions of the 16S rRNA gene was employed to quantify the overall bacterial population (30). The qPCR experimental procedure was detailed in our prior investigation (26). Each reaction was executed in triplicate to ensure accuracy and reliability.

**TABLE 2:** Primers used in this study

Cell	Sequence (5′−3′)	Amplicon length (bp)	Reference
Primer 1 (*B. longum* ZS-8)	F: GTACTGGAATCTGGTGGGGC R: CCTCGTCGCCTGTCTCAATT	115	This study
Primer 2 (*B. longum* ZS-8)	F: TACTGTGTGTTCGACGACGG R: TATGCCCATCGACTCATGGC	127	This study
Primer 3 (*B. longum* ZS-8)	F: GAATGCGCGGTATGTTCCAC R: AAGCAGATTGGTTCGCCGTA	119	This study
Primer 4 (*B. longum* ZS-8)	F: CCGACTGGCTGGAATTCGAT R: CCGTTTCGTCTCCACGTACA	167	This study
*Lactobacillus* spp.	F: TGGATGCCTTGGCACTAGGA R: AAATCTCCGGATCAAAGCTTACTTAT	89	30
*Bifidobacterium* spp.	F: GCGTGCTTAACACATGCAAGTC R: CACCCGTTTCCAGGAGCTATT	105	29
16S rRNA	F: AAACTCAAAKGAATTGACGG R: CTCACRRCACGAGCTGAC	1,490	
16S rRNA V6	F: AAACTCAAAKGAATTGACGG R: CTCACRRCACGAGCTGAC	136	31

### Quantification of viable ZS-8 in pure cultures and human feces

Feces were collected and diluted as described in our previous study (23). To assess the presence of ZS-8 in native feces, we analyzed 500 µL of diluted fecal samples from three healthy volunteers (which were not spiked with ZS-8) using qPCR with primers specific to ZS-8. Subsequently, 3.0 mL of diluted fecal samples was spiked with either viable or heat-killed ZS-8 and divided into six equal aliquots. Three aliquots were used as the control group (without lyPMAxx treatment), while the remaining three were subjected to lyPMAxx treatment. The viable counts of ZS-8 were determined using plate-counting.

For the quantitative detection of ZS-8 in fecal samples, a standard curve was generated by correlating Ct values with viable populations. Briefly, viable ZS-8 was spiked into three diluted fecal samples at concentrations ranging from 10^4^ to 10^10^ CFU per gram of feces. These concentrations were re-determined by counting colony-forming units (CFU) on plates for each dilution series of the standard points (31). Subsequently, after homogenizing and treating the spiked stool samples with PMAxx, we extracted and analyzed DNA by qPCR using the F3 primer pairs.

### Human subjects

This study was a 42-day sequential cohort trial involving 41 healthy adult volunteers (with two dropouts) to evaluate the viability and colonization of the administered ZS-8 in the human gut and its influence on the microbiota community. All participants provided signed informed consent during the screening stage.

Key inclusion criteria were male or female subjects in general good health, aged 18 to 59 years. Exclusion criteria included lactation or pregnancy, presence of blood in stool, history of antibiotic use within 3 months before the intervention, history of bowel cleansing, ingestion of probiotics within 1 month before the intervention, history of drug or alcohol abuse, use of immunosuppressive drugs, and digestive system diseases that significantly impact the study, such as inflammatory bowel disease (IBD) and infectious gastrointestinal diseases. Additionally, participants were prohibited from taking any drugs or health products/foods that could cause diarrhea or alter the intestinal flora during the trial, including laxatives, both Western and Chinese medicine, antibiotics, probiotics, and yogurt.

The volunteers ingested different preparations of ZS-8 for 14 days. Subjects in groups 1 and 2 ingested lyophilized powder of ZS-8 [with or without fructo-oligosaccharide (FOS)] at a daily dose of 1.0 × 10^10^ CFU. FOS was included to promote overall gut health by serving as a prebiotic substrate for beneficial bacteria. Subjects in groups 4 and 5 received ZS-8 MLSC at a daily dose of 1.0 × 10^10^ CFU (with or without FOS), respectively. Subjects in group 3 received ZS-8 MLSC at a daily dose of 1.0 × 10^9^ CFU. Follow-up visits were scheduled every 1–2 weeks for approximately 1.5 months. Stool samples were collected at the baseline visit (day 0), intervention visit (days 7 and 14), and follow-up visit (days 21, 28, and 42). The methods of collection, dilution, and pretreatment (with or without lyPMAxx) of fresh fecal samples are as described in our previous study (25). A summary of demographic and baseline subject characteristics and dosages is reported in Table 3. All subjects were required to complete an electronic stool diary from screening until the end of the study. Stool consistency was measured using the Bristol Stool Form Scale (BSFS), and stool frequency was measured by the weekly mean complete spontaneous bowel movements (CSBMs).

**TABLE 3:** ZS-8 dosing summary and demographics

	Group 1	Group 2	Group 3	Group 4	Group 5
Dosing					
Daily dose (g)	0.4 g	0.4 g + 10 g FOS	0.34 g	3.36 g	3.36 g + 10 g FOS
Daily dose (CFU)	2.00E + 10	2.00E + 10	2.00E + 9	2.00E + 10	2.00E + 10
Days administered	14	14	14	14	14
Total dose (CFU)	2.80E + 11	2.80E + 11	2.80E + 10	2.80E + 11	2.80E + 11
Characteristics					
Number	8	8	8	9	8
Age (years)	27.25 (11.50)	36.75 (15.01)	32.13 (13.21)	29.33 (9.80)	33.25 (6.92)
Females, *n* (%)	3 (37.5）	3 (37.5）	3 (37.5）	4 (44.4）	3 (37.5）
BMI (kg m^−2^)	22.73 (1.84)	23.94 (4.12)	23.54 (3.81)	22.84 (4.61)	24.36 (2.79)

### Bacterial isolates

The fresh feces were collected and pretreated (without lyPMAxx) as described in our previous study (25), then serially diluted 10-fold in phosphate-buffered saline (PBS) containing 0.05% l-cysteine hydrochloride. The 10^6^-fold diluted samples were spread onto selective agar plates and incubated in an anaerobic chamber (H2/CO2/N2, 10:10:80, vol/vol) at 37°C for 48 hours. Media used for isolation and enumeration were LBS agar for *Lactobacillus* and MRS medium for *Bifidobacterium* (32, 33). Certain isolates were further identified by 16S rRNA gene sequence analysis. Genomic DNA was extracted using a Takara bacteria genomic DNA extraction kit (Takara, Kyoto, Japan) according to the manufacturer's instructions. Full-length 16S rRNA gene amplification, library preparation, and sequencing were performed at Tsingke (Guangzhou, China) using an ABI 3730 DNA sequencer. Searches of current nucleotide databases were performed with the BLAST algorithm from NCBI (http://www.ncbi.nlm.nih.gov). A similarity of >99% in 16S rRNA gene sequence was used as the criterion for identifying an isolate at the species level.

### Construction of amplicon library of V3-V4 region of 16S rRNA and sequencing

Fecal DNA was amplified using universal primers targeting the V3-V4 region of 16S rRNA genes (338F: 5′-ACTCCTACGGGAGGCAGCA-3′ and 806R: 5′-GGACTACHVGGGTWTCTAAT-3). The PCR reaction was carried out in a PCR system (ABI 2720, Carlsbad, CA, USA) as follows: initial denaturation at 96°C for 5 min, followed by 25 cycles of denaturation at 96°C for 30  seconds, annealing at 50°C for 30 seconds, and elongation at 72°C for 30 seconds, with a final extension step at 72°C for 5 minutes. The PCR products were visualized on a 1.5% agarose gel and purified by the QIAquick Gel Extraction Kit (QIAGEN; cat# 28706). Purified PCR products were then used to construct a metagenomic library with the Illumina TruSeq Sample Preparation Kit (Illumina, San Diego, CA, USA). The V3-V4 amplicons were then sequenced in paired-end mode on the Illumina MiSeq platform (Illumina, San Diego, CA, USA) according to the manufacturer's instructions.

### Bioinformatic analysis of amplicon sequencing

Quantitative Insights Into Microbial Ecology version 2 (QIIME2, version 2022.2) was used to process raw sequencing data into an ASV table (34). Initial quality filtering of raw reads was performed based on sequence quality scores using the qiime quality-filter q-score command with default settings (35). The Deblur plugin was used to filter the sequencing reads and construct an ASV feature table (36). Taxonomy for the ASVs was assigned against the Greengene Database. A median of 61,318 reads was obtained per sample (Table S7), followed by rarefaction to 38,275 reads according to the rarefaction curve to ensure even sampling depth (Table S8). Alpha and beta diversity metrics were calculated by the vegan package of the R software (v4.3.1). PCoA plots were generated using the ggplot2 package of the R software (v4.3.1).

### Statistical analysis

Statistical analysis was conducted using GraphPad Prism 8.21 software (La Jolla, CA, USA). Paired *t*-tests were used to analyze the total 16S rRNA gene copy numbers and the counts of viable ZS-8, *Lactobacillus*, and *Bifidobacterium* in fecal samples. The Wilcoxon test was used to analyze the alpha diversity of the fecal microbiota (*P  <  0.05, **P  <  0.01, ***P  <  0.001, and ****P  <  0.0001). Results were expressed as means ± the standard error of the mean (SEM).

## RESULTS

### Design and validation of strain-specific qPCR

**Fig 1:**
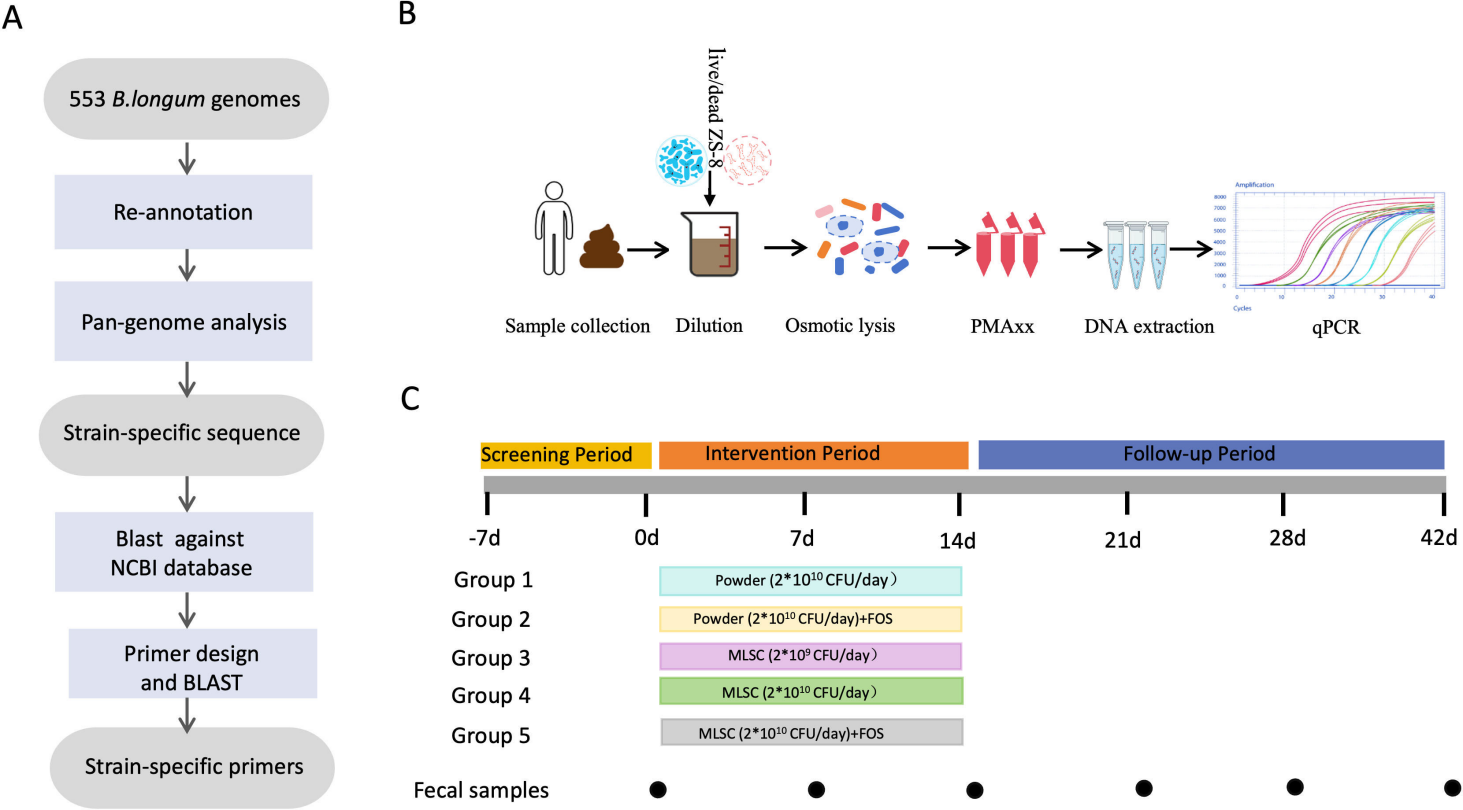
Workflow and research pipelines for ZS-8. (**A**) The workflow for designing strain-specific primers for ZS-8 through comparative genomics. (**B**) The research pipeline for quantifying viable ZS-8 in fecal samples using the lyPMAxx method. (**C**) Study design and timeline for sample collection from healthy adult participants.

The ZS-8 strain was isolated from healthy infant feces, and *B. longum* is broadly distributed across subjects of various ages (13). Therefore, distinguishing ingested strains from native gut strains is crucial. In this study, we developed strain-specific primers for ZS-8. The pipeline used to construct strain-specific primers is illustrated in Fig. 1A. First, we performed whole-genome sequencing of the ZS-8 strain and assembled the sequencing data using SPAdes (37) to obtain a draft genome of the strain. Subsequently, we re-annotated 552 publicly available *B. longum* genomes and the ZS-8 genome using Prokka. Then, we employed a Roary-based pan-genome analysis to identify unique gene markers exclusive to the ZS-8 strain, which are absent from all other sequenced strains of this species and subspecies. Based on gene sequences, we conducted a gene presence/absence analysis, which identified four unique genes specific to ZS-8. Additionally, we utilized a website-based nucleotide BLAST tool against the NR/NT database of NCBI to confirm that these sequences were not found in any other representative microbes. Finally, HPHHGGJE_01475, HPHHGGJE_01548, HPHHGGJE_01549, and HPHHGGJE_01550 were selected as the target DNA sequences for strain-specific quantification of ZS-8 (Table 1).

Four primer pairs were specifically designed for the ZS-8 strain based on its unique gene sequences (Table 2). The product sizes were 115, 127, 119, and 167 bp, respectively. To validate the specificity of these primers, we tested them against various microbial strains, including seven other *B. longum* strains, six strains of other *Bifidobacterium* species, seven *Lactobacillus* strains, 18 bacteria representing members of the human gut microbiota, and eight human fecal samples (native or spiked with ZS-8) (Table S1). The primers specifically amplified only ZS-8, with no amplification observed in nontarget microbes (Table S2). These results confirmed that the four pairs of primers were unique to the ZS-8 strain and could be used to quantify ZS-8 in subsequent experiments. Primer 1 was selected for use in this study.

### PMAxx-qPCR effectively quantified viable ZS-8 in human feces

**Fig 2:**
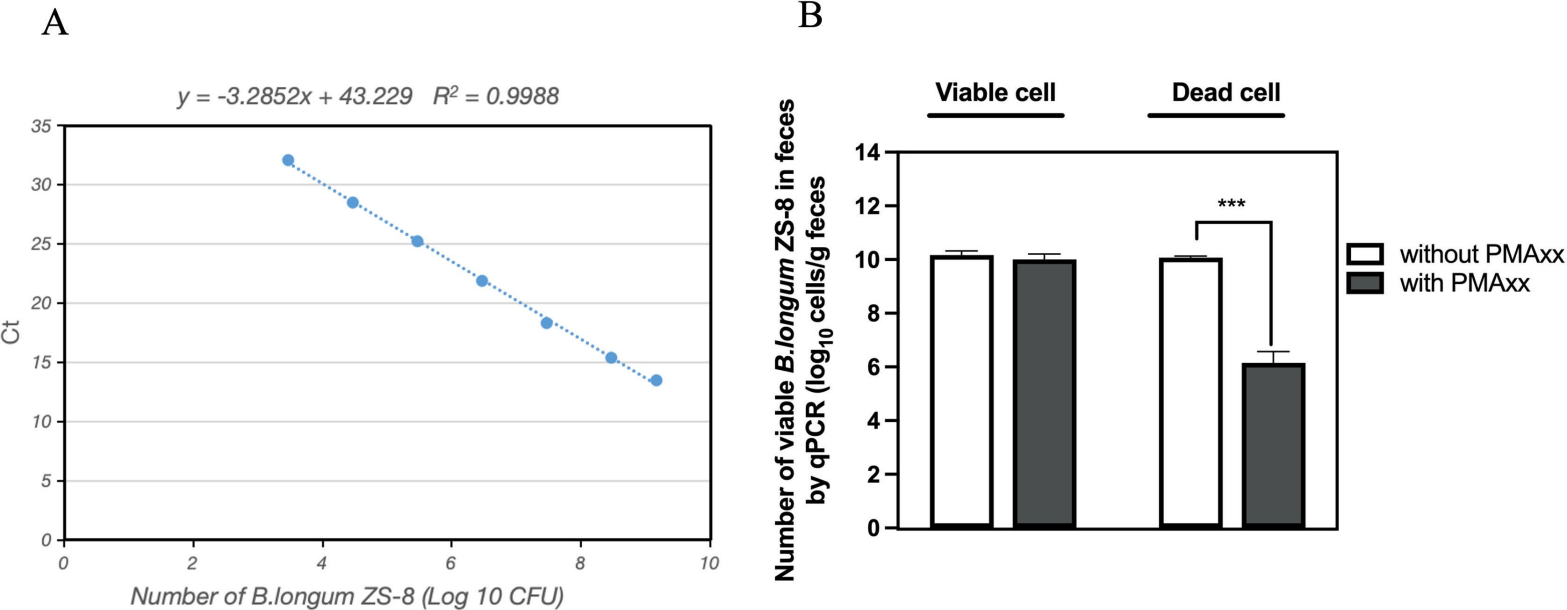
Use of qPCR with PMAxx to determine the number of ZS-8 viable cells in human feces. (**A**) Correlation between the number of viable ZS-8 strain added to faecal samples and that determined by qPCR with PMAxx treatment. (**B**) Effect of PMAxx treatment on qPCR amplification of viable and heat-killed ZS-8 cells in human feces. ***, *P* < 0.001. Data are the mean ± SD (from three independent experiments).

ZS-8 was not detected in the native feces of the three healthy volunteers (Table S3). Subsequently, viable ZS-8 was introduced into three fecal samples in amounts ranging from 10^4^ to 10^9^ CFU per gram of feces. We analyzed the correlation between the number of live ZS-8 cells and the Ct value obtained via strain-specific qPCR. The standard curves exhibited good linearity over a 4-log range (10^3^–10^7^ CFU/qPCR reaction, with an R² value of 0.9932). The regression equation for ZS-8 was as follows: Ct = −3.0415*lg CFU + 43.043 (Fig. 2; Table S4). Collectively, the primer pairs demonstrated specificity for the ZS-8 strain, and PMAxx-qPCR accurately quantified viable target *B. longum* cells in fecal samples.

The viability of probiotics upon reaching the intestine is crucial for their beneficial effects (38). However, the presence of gastric acid, digestive enzymes, and bile salts presents significant challenges to the oral probiotics. To accurately assess probiotic viability in the intestine, it is essential to establish a reliable method. In this study, we employed a strain-specific PMAxx-qPCR method. Our first step was to evaluate the feasibility of this method, particularly its ability to distinguish between dead and live cells, ensuring that only viable cells are quantified. We spiked fecal samples with equal amounts of live or dead ZS-8 cells. When live ZS-8 was added, the number of ZS-8 cells detected by PMAxx-qPCR was comparable to that detected by qPCR without PMAxx treatment (Fig. 2B; Table S5). Conversely, when heat-killed ZS-8 was spiked into the feces, PMAxx-qPCR successfully removed more than 99% of the dead cells (Fig. 2B; Table S5). These results demonstrate that PMAxx-qPCR effectively eliminates dead ZS-8 cells and accurately quantifies viable ZS-8 in human feces.

### Transient survival of ZS-8 in healthy volunteers

**Fig 3:**
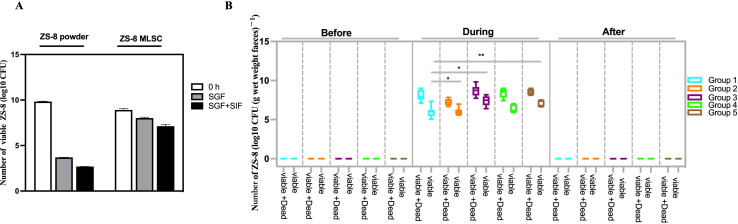
Survival of ZS-8 (free and MLSC) *in vitro* and *in vivo*. (**A**) The number of viable ZS-8 in SGF and SGF + SIF. (**B**) The number of viable ZS-8 in the human feces before, during, and after the interventions.

To evaluate the tolerance and survival of ZS-8 *in vitro*, we investigated its viability in SGF and SIF for both free and MLSC forms by plate-counting. The results showed that MLSC significantly improved the survival rate of ZS-8 in both SGF and SIF (*P* < 0.001; Fig. 3A; Table S6). Specifically, 12.34% and 1.65% of ZS-8 strains remained viable after digestion in SGF (pH 1.2, 2 h) or SGF followed by SIF (pH 6.8, 4 h) in the MLSC group, whereas almost no viable cells were detected in the powder group after SGF alone. This indicates that non-encapsulated ZS-8 could not survive at low pH, while MLSC of ZS-8 exhibited significantly greater resistance to both SGF and SIF.

Building on these findings, we then conducted an *in vivo* study to evaluate the survival and efficacy of ZS-8 in a real gastrointestinal setting. Forty-one healthy volunteers participated in this study, receiving either MLSC or lyophilized powder of ZS-8 at lower doses (2.0 × 10^9^ CFU/day) or higher doses (2.0 × 10^10^ CFU/day), with or without FOS for 14 days (Table 3; Fig. 1C). Two subjects, being noncompliant with the protocol, were excluded from further analysis. PMAxx-qPCR was employed to quantify the number of viable ZS-8 cells in the feces of participants after consuming the probiotics for 14 days. Before ingestion, ZS-8 strains were not present in the native feces of any of the healthy volunteers as validated by qPCR. On the last day of the 2 week intervention, 1.53% and 6.90% of viable ZS-8 were detected in the lyophilized powder and MLSC groups, respectively (Fig. 3B; Table 4; Table S16). The number of live ZS-8 cells in the feces of the MLSC groups (groups 3 and 4) was 6.29 and 3.90 times higher than in the lyophilized powder group (group 1, *P* < 0.005). Additionally, the survival rate of the MLSC + FOS group (group 5) was 3.79 times higher than that of the powder + FOS group (group 2), although this difference was not statistically significant in fecal samples (Fig. 3B; Table 4; *P* > 0.05). Comparing the groups with and without FOS, no significant improvement in ZS-8 survival was observed. The results suggest that the delivery method played a more crucial role than FOS in enhancing ZS-8 survival. Overall, MLSC significantly improved ZS-8's tolerance in the gastrointestinal environment, resulting in better survival rates compared to lyophilized powder. This highlights the practical advantages of MLSC technology for oral probiotic delivery.

**TABLE 4:** Group statistics of viable and total ZS-8 counts in feces on intervention day 14

Group	log10 CFU (live + dead)/g feces	log10 CFU (live)/g feces	Viable percent (%)
1	8.00 ± 0.67	6.00 ± 0.71	1.62 ± 1.16
2	8.39 ± 0.59	6.35 ± 0.50	1.41 ± 1.43
3	7.23 ± 0.43	6.03 ± 0.50	9.62 ± 9.46^*a*^
4	8.71 ± 0.67	7.43 ± 0.65	5.96 ± 2.93^*a*^
5	8.52 ± 0.37	7.00 ± 0.44	5.35 ± 7.04

^*a*^ *P* < 0.005 vs group 1 (6.29- and 3.90-fold higher in groups 3 and 4, respectively).

### The gut microbiota remained stable after ZS-8 administration

**Fig 4:**
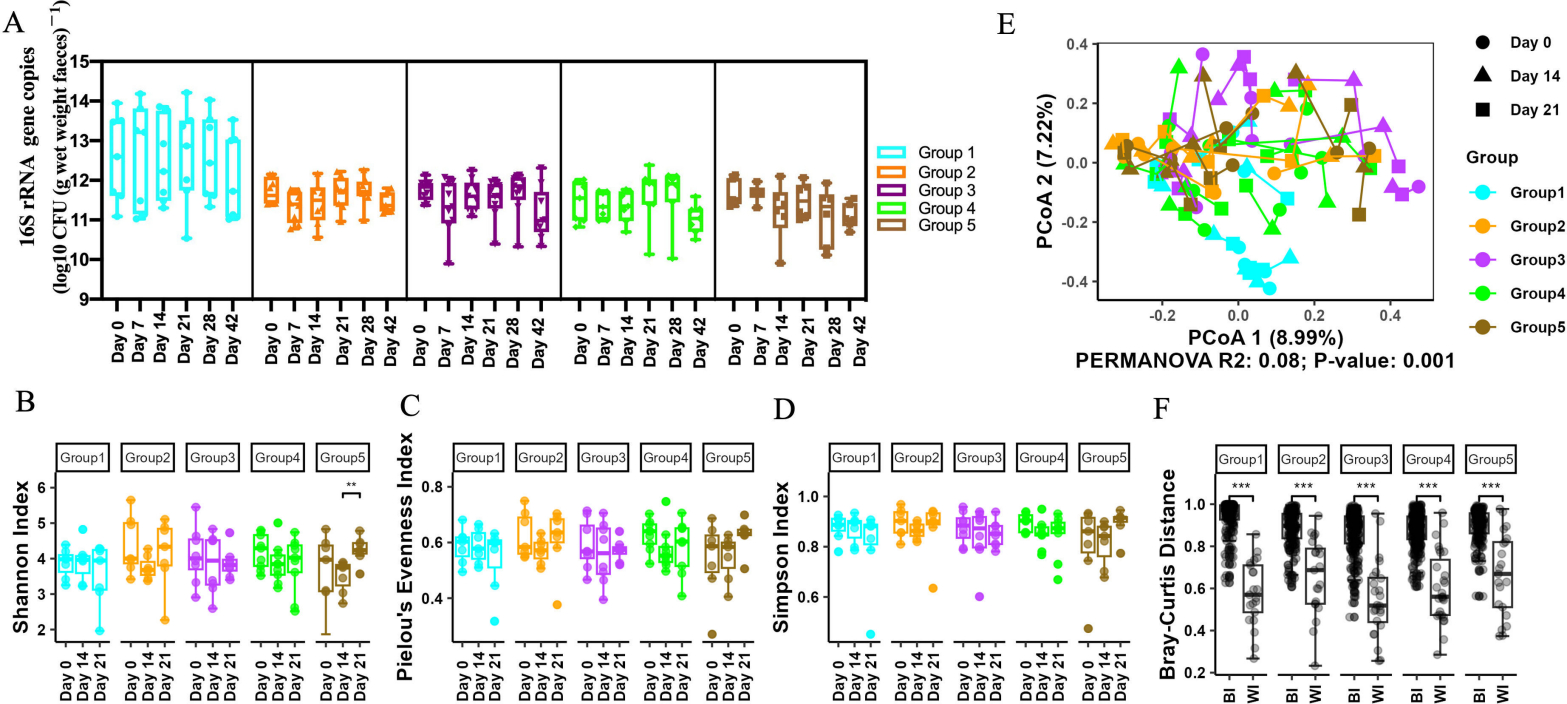
Microbiota dynamics with ZS-8. (A) 16S rRNA gene copy numbers across interventions. (B, C and D) Variation of alpha diversity (Simpson/Shannon/Pielou). (E) PCoA of fecal Bray-Curtis distances. (F) Inter-individual (BI) vs intra-individual (WI) dissimilarity.

Viable gut bacterial loads following ZS-8 intervention were quantified using PMAxx-qPCR targeting the 16S rRNA V6 gene. The total microbial biomass across groups 1–5 ranged from 10^10^ to 10^14^ copies per gram of feces, with no significant differences observed between or within groups (Fig. 4A). Subsequently, we evaluated the effect of ZS-8 supplementation on the gut microbiota on days 0, 14, and 21, representing the periods before, during, and after the intervention, respectively. There were no significant changes in Simpson index, Shannon index, or Pielou's evenness between time points in most of the groups (except *P* < 0.01 between days 14 and 21 of group 5 for Shannon index; Fig. 4B through D), suggesting relatively stable alpha diversity throughout the 21-day period. Similarly, a principal-coordinate analysis (PCoA) also revealed that the samples from the same group did not cluster together before and after the intervention (Fig. 4E). Further statistical analysis of the Bray-Curtis distance between intra-individual and inter-individual samples showed that the fecal microbiota structure during the intervention and follow-up periods remained comparable to the baseline. This suggests that the gut microbiota remained relatively stable after ZS-8 administration (Fig. 4F), and the impact of ZS-8 was less pronounced compared to the variability observed between different hosts (*P* < 0.005; Fig. 4F).

**Fig 5:**
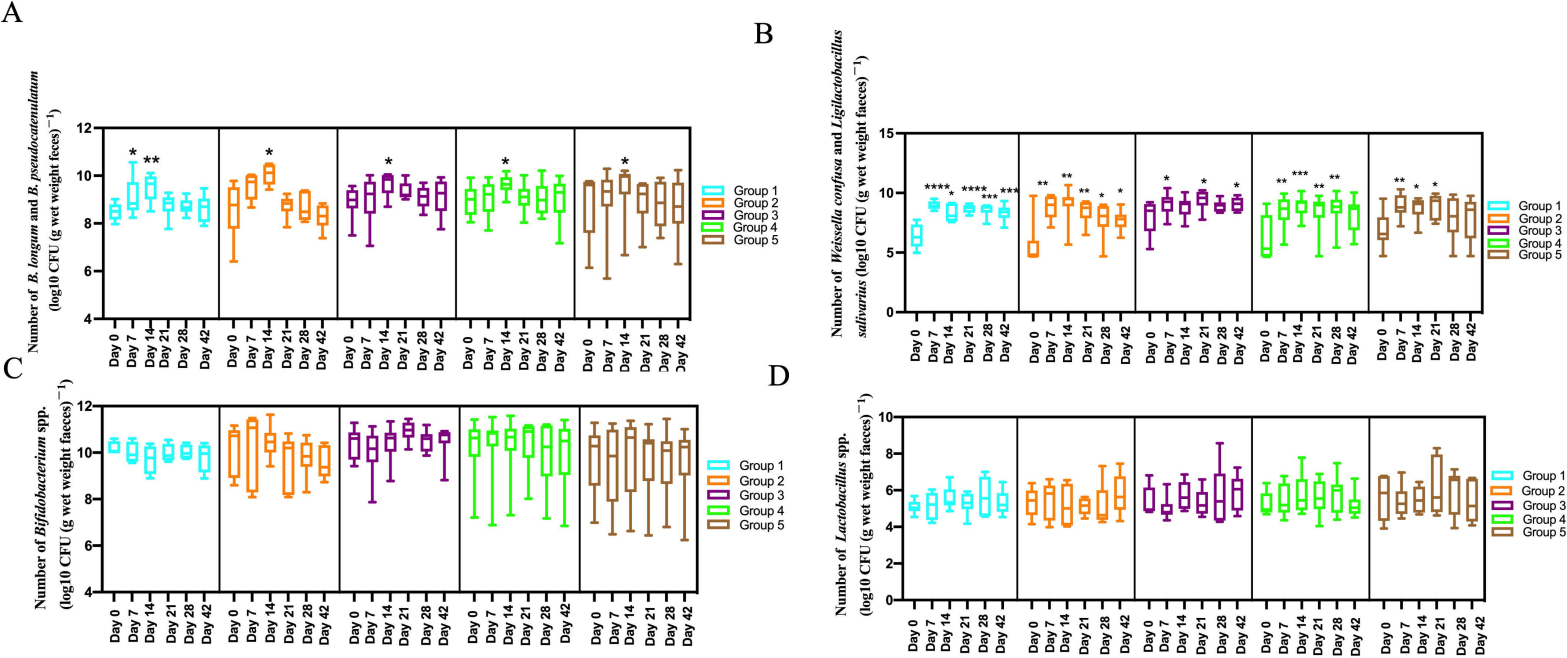
Change in the number of viable beneficial bacteria during/after interventions. (**A**) The number of viable *Bifidobacterium longum* and *Bifidobacterium pseudocatenulatum* during/after interventions counted by plate-counting. (**B**) The number of viable *Weissella confusa* and *Ligilactobacillus salivarius* during/after interventions counted by plate-counting. (**C**) The number of viable *Bifidobacterium* spp. during/after interventions by PMAxx-qPCR. (**D**) The number of viable *Lactobacillus* spp. during/after interventions by PMAxx-qPCR.

In this study, PMAxx-qPCR was used to evaluate changes in the quantities of total viable *Bifidobacterium* and *Lactobacillus* spp. in fecal samples from subjects taking ZS-8. There were no significant differences in the quantities of total viable *Bifidobacterium* and *Lactobacillus* between pre- and post-interventions (Fig. 5C and D). Meanwhile, a culture method with MRS (for *Bifidobacterium*) or LBS agar (for *Lactobacillus*) was employed to assess changes in these two beneficial bacterial taxa. At baseline, the number of *Bifidobacterium* clones in the fecal samples of all subjects was 8.51 ± 0.87, with no significant differences among groups. The CFU counts of *Bifidobacterium* colonies increased during the intervention period (days 7 and 14) compared to baseline, with a significant improvement on the 14th day of intervention across all groups (9.63 ± 0.69, *P* < 0.05; Tables S10 and S11). After the intervention, the quantity of culturable *Bifidobacterium* decreased to baseline levels within 1–3 weeks for groups 1–5 (Fig. 5A). Given the increased CFU counts of *Bifidobacterium* colonies, we suspected that the increased CFU counts of *Bifidobacterium* colonies might come from ZS-8, prompting further identification of the isolated strains. We obtained 48, 12, and 50 isolates from the fecal samples of volunteers 101, 107, and 202, respectively, and subsequently identified them using 16S rRNA gene sequencing. Almost all the 110 isolates belonged to *B. longum* or *Bifidobacterium pseudocatenulatum* (Table S14). Unexpectedly, ZS-8 strain-specific qPCR confirmed that all *B. longum* isolates were not ZS-8 but rather indigenous bacteria present in the gut. This suggests that the specific indigenous *B. longum* proliferated following the administration of exogenous ZS-8. We propose that the oral administration of ZS-8 may have altered the gut microenvironment, thereby promoting the growth of certain beneficial indigenous bacteria. Similarly, the number of culturable *Lactobacillus* colonies in the fecal samples of all subjects was 6.68 ± 1.69 at baseline with no significant differences among the five groups (Fig. 5B; Tables S12 and S13). The number of culturable *Lactobacillus* significantly increased on the 7th and 14th days following the ZS-8 intervention (*P* < 0.0001). This increase was sustained for 1–3 weeks post-intervention compared to the baseline period (Fig. 5B; Tables S12 and S13). The overall data for the six time points are as follows: 0th day (6.68 ± 1.69), 7th day (8.80 ± 0.97), 14th day (8.76 ± 1.01), 21st day (8.78 ± 1.08), 28th day (8.39 ± 1.26), and 42nd day (8.25 ± 1.22). Figure 5B presents the changes in *Lactobacillus* numbers for each group individually over these time points. From volunteers 101, 202, 207, 305, 307, and 508, we identified 10, 10, 2, 12, 9, and 10 isolates, respectively. Using 16S rRNA gene sequencing analysis, we identified nearly all 53 isolates as *Weissella confusa* or *Ligilactobacillus salivarius* (Table S15).

### ZS-8 induced no significant changes in the CSBM and BMI in healthy subjects

Forty-one healthy volunteers participated in the study, with ages ranging from 27.25 ± 11.50 to 36.75 ± 15.01 years, male-to-female ratios of 5:3 or 5:4, and BMI ranging from 22.73 ± 1.84 to 24.36 ± 2.79. There were no significant differences in demographic and baseline characteristics among the groups (*P* > 0.18; Table 3). At baseline, there were no significant differences observed among the five groups in terms of BMI, their mean weekly frequency of complete spontaneous bowel movements (CSBMs), stool consistency (Bristol Stool Form Scale, BSFS), and pH levels. After 14 days of intervention, these indicators remained similar among the five groups, with no significant differences (Table 5). The stability of the volunteers' characteristics following the intervention indicates that the administration of ZS-8 is safe. Overall, the healthy volunteers maintained intestinal homeostasis and exhibited minimal response in defecation patterns following ZS-8 intervention.

**TABLE 5:** Changes in fecal indicators across different groups over 14 days

Dosing	Group 1	Group 2	Group 3	Group 4	Group 5
BMI change	0.13 ± 0.32	0.07 ± 0.58	0.16 ± 0.19	0.17 ± 0.28	0.15 ± 0.44
Change in weekly mean frequency of CSBMs	0.13 ± 0.35	0.17 ± 0.41	0.13 ± 0.35	0.44 ± 0.73	0.00 ± 0.00
Change in stool consistency (BSFS) from the baseline	1.14 ± 0.90	1.00 ± 0.00	0.75 ± 0.89	1.00 ± 0.53	1.29 ± 1.60
pH change	0.46 ± 0.35	0.69 ± 0.46	1.52 ± 0.91	0.61 ± 0.38	0.67 ± 0.44

## DISCUSSION

*Bifidobacterium* spp. have been shown to possess the ability to shift the predominant bacteria of the gut microbiota by promoting beneficial bacteria and inhibiting pathogens (3). However, the impact of *Bifidobacterium* on healthy volunteers is seldom investigated (39). Our study demonstrates that 1.53–6.90% of ZS-8 cells remain alive after delivery through the gastrointestinal tract in healthy volunteers, as determined by PMAxx-qPCR, and no viable cells were detected on a week post-administration, indicating that ZS-8 did not colonize in healthy GITs. Nevertheless, ZS-8 administration significantly increased some culturable native *B. longum* and *B. pseudocatenulatum* populations during the intervention period of 1–2 weeks. This suggests that, despite the transient presence of ZS-8 in the gut, it can still have a positive impact on the gut microbiota by increasing the abundance of specific beneficial native bacteria in the short term. The main objective of this study was to evaluate the short-term survival and safety of this strain. A 14-day short-term intervention was conducted, and therefore, data on the long-term effects of ZS-8 are lacking. It warrants extending the intervention period to comprehensively assess the long-term colonization and sustained therapeutic effects of ZS-8 in the future.

*B. longum* is one of the most prevalent *Bifidobacterium* species in the adult gut (13). To differentiate exogenously administered *B. longum*, particularly the ZS-8 strain, from native strains, we developed a strain-specific method for identifying the ZS-8 strain in this study. Maintaining the viability and activity of probiotics in the gut after ingestion is crucial for them to promote host intestinal health (1). Thus, we aimed to investigate the activity of ZS-8 in the human gut. We established a set of PMAxx-qPCR assays to identify and quantify viable ZS-8 cells. Strain-specific DNA sequences of ZS-8 were identified through pan-genome analysis, and qPCR primers were then designed based on these unique markers. The use of PMAxx-qPCR and strain-specific primers adds novelty and ensures accurate quantification of viable probiotics. This approach enabled us to detect *B. longum* strains in human feces with strain-level resolution. Compared to traditional culture-based methods, our method offers superior sensitivity and specificity for distinguishing exogenous strains from native microbiota (24), reducing the labor and cost required for cultivation and identification.

The most common delivery route for probiotics is oral administration, with tablets or capsules being the primary delivery forms. However, a concern with tablets is the heat generated during compression, reaching temperatures up to 60°C, which can negatively affect the viability of the probiotics (40, 41). Other delivery systems, such as microcapsules and beads, have been shown to significantly enhance the survival of viable bacteria during storage and subsequent delivery to the intestine (42, 43). MLSC is a seamless encapsulation technology that encapsulates live bacteria in the innermost layer. To assess the efficacy of a probiotic-loaded delivery system, it is crucial to evaluate the probiotic's tolerance during gastrointestinal transit. In this study, we conducted both *in vitro* and *in vivo* assessments, providing robust insights into the viability of ZS-8 and its impact on host health. SGF and SIF are commonly used to evaluate the viability of probiotics *in vitro* and are typically prepared based on the United States Pharmacopeia recipe (44). In our study, we assessed the survival of ZS-8 in a simulated GIT environment using these methods. Results showed that the MLSC containing ZS-8 exhibited significantly higher survival rates, with a five-log unit increase compared to free cells (lyophilized powder) following a 2 hour incubation in SGF at pH 1.2 (Fig. 3A; Table S6). *In vitro* digestion models cannot accurately replicate the human gut environment, and the absence of biokinetics *in vitro* methods can lead to data misinterpretation (44, 45). Mouse models are frequently used to validate probiotics *in vivo*, but the intestinal physiology differs significantly between mice and humans. Only 4% of bacterial genes are similar between human and murine intestines (46). Therefore, we conducted a direct *in vivo* assessment in humans. We then evaluated the probiotic characteristics of ZS-8 using PMAxx-qPCR in healthy volunteers. Our findings revealed that ZS-8 successfully traversed the gastrointestinal tract following a 14-day ZS-8 intervention in healthy adults, and 1.53% and 6.90% of viable ZS-8 cells were detected in the lyophilized powder and MLSC groups, respectively (Fig. 3B). This finding is consistent with previous research. In a study involving 12 healthy subjects who ingested 10^10.3^–10^11^ CFU/day *Bifidobacterium bifidum* BF-1 for 28 days, 10^6.2^ viable BF-1 cells and 10^7.6^ total cells per gram of feces were detected. The survival rate of BF-1 in the colon was determined to be 3.98% (1). Meanwhile, our results indicate that MLSC improves the gastrointestinal tolerance of ZS-8 both *in vitro* and *in vivo*. Liu et al. reported that microgels enhanced the viability of *Lactobacillus plantarum* MA2 both in *vitro* and murine models (47). The protective function of probiotics on host health often depends on the presence of viable cells (48). One study reported that live cells of *Lactobacillus casei* PLA12 and PLA5 exhibited higher cholesterol removal rates (47.70  µg/mL) compared to dead cells (4.74  µg/mL), suggesting that cholesterol adherence to bacterial cell membranes is growth-dependent (49). Similarly, Liu et al. showed that encapsulated *Lactobacillus rhamnosus* GG (LGG) was more effective at suppressing gut inflammation in mice compared to the free form (47). A clinical study indicated that supplementation with high doses (≥5 billion CFU/day) of LGG was more effective in reducing the incidence of antibiotic-associated diarrhea than lower doses (<5 billion CFU/day) (50). The MLSC formulation technology uses multi-layer encapsulation to protect sensitive ZS-8 during its passage through the gastrointestinal tract. By preserving probiotic viability in the intestines, the MLSC technology may enhance the health-promoting effects of ZS-8.

Our findings indicate that 1.53–6.90% of ZS-8 cells remain viable during the 2 week administration period. However, no viable cells were detected in 1 week post-administration (Fig. 3B), suggesting that ZS-8 is excreted within a few days after ingestion in healthy human feces and does not colonize the gastrointestinal tracts of healthy GITs. Dsouza et al. reported that VE303 strains, a type of live biotherapeutic product (LBP), optimally colonized the healthy volunteers with vancomycin pretreatment but could not colonize without antibiotics (51). Button et al. indicated that, in healthy subjects administered with only *Bifidobacterium longum* subsp*. infantis* (*B. infantis*) for 8 days without antibiotic pretreatment, the average of *B. infantis* signal dropped to below the limit of detection by day 15, 1 week after *B. infantis* dosing had ended (52). This phenomenon may be due to the colonization resistance exhibited by the healthy microbiota. As colon microbes evolve and adapt, numerous environmental niches within the human gut become filled with indigenous microbiota, forming a formidable barrier to subsequent colonization by invading foreign microbes (53). The antibiotic administration results in the loss of species, microbiota dysbiosis, and an increased ability to introduce new species (51). The absence of long-term colonization of ZS-8 in the healthy human gut may be attributed to the high stability of the gut microbiota in healthy individuals (53). Although our study did not observe persistent colonization, ZS-8 can survive effectively in the gut in the short term and potentially promote the growth of indigenous beneficial bacteria. However, this effect is only evident during the period of probiotic administration, highlighting its dependence on continued probiotic intake. The short-term survival and activity of ZS-8 underscore its potential as a probiotic, particularly in the treatment of acute gut health issues, such as diarrhea associated with gut microbiota dysbiosis (54).

The present study revealed no significant changes in alpha diversity following ZS-8 administration. This transient presence, coupled with the inability to colonize, may explain the lack of sustained effects on the gut microbiota. In a study with 150 healthy adults consuming yogurt fortified with *Bifidobacterium animalis* subsp*. lactis* BB-12, no changes in microbial community richness were observed (15). Another study on the gut microbiota of 37 healthy adults, sampled 2 to 13 times over a span of up to 296 weeks, revealed that their microbiota was highly stable, with 60% of strains persisting over the course of 5 years (55). This stability is attributed to the remarkably stable microbiota of healthy adults (53). These findings enhance our understanding of the role of probiotics in healthy individuals. The gut microbiota of healthy individuals exhibits significant colonization resistance (53). Supplementation of probiotics in such populations may not be necessary, as probiotics can only survive transiently in the healthy gut without achieving long-term colonization, potentially limiting their efficacy.

In this study, the microbiome composition varied more by subject than by intervention, consistent with previous findings. For instance, a synbiotic intervention with *Bifidobacterium*, *Lactobacillus*, and inulin in healthy volunteers showed that microbial community composition clustered by subject rather than by baseline or post-intervention states (39). Despite this individual variability, significant changes in specific microbial populations were observed following the intervention. Notably, there were increases in the numbers of culturable *Bifidobacterium* and *Lactobacillus* species in our study. Further qPCR analysis showed that the main increase was indigenous beneficial bacteria. This phenomenon was consistent across both groups, with and without FOS, suggesting that *Bifidobacterium*, rather than FOS, drove the increase in indigenous beneficial bacteria in gut microbiota. Rubin et al. found that 12 out of 15 healthy individuals who received synbiotics (*Bifidobacterium*, *Lactobacillus*, and inulin) had an increased relative abundance of native *Bifidobacterium* (39). In another study involving 150 healthy individuals who consumed yogurt for 2 weeks, an increase in the relative abundance of the *Bifidobacterium* genus was observed (15). The observed increases in indigenous *Bifidobacterium* and *Lactobacillus* in this study may be linked to cross-feeding interactions (56, 57). This metabolic exchange contributes to the stability and growth of microbial communities, which has been widely observed in *Bifidobacterium* spp. (58). For instance, *Bifidobacterium bifidum* PRL2010 has been shown to degrade mucin and release sugars that can be utilized by *Bifidobacterium breve* UCC2003 (59). Similarly, acetate produced by *B. longum* BB536 during carbohydrate fermentation can stimulate the growth of butyrate-producing bacteria in the colon and promote the production of butyrate *in vitro* (60). *B. longum* tends to utilize short-chain inulin, thereby cooperating with *Lactobacillus paracasei* to jointly degrade long-chain inulin and produce fructose, lactate, and acetate, demonstrating a cross-feeding interaction between these two strains (57). These findings suggest that certain species of *Bifidobacterium*, through their metabolic products, may support the growth of other beneficial indigenous bacteria via cross-feeding. Previous studies have demonstrated that the ZS-8 strain produces large amounts of lactate and acetate during carbohydrate fermentation (Table S17), and these metabolites may be utilized by indigenous gut microbes, leading to their proliferation. Future research could explore the metabolites involved in these cross-feeding interactions to better understand the mechanisms driving the observed increases in indigenous *Bifidobacterium* and *Lactobacillus*.

### Conclusion

We developed PMAxx-qPCR method with high sensitivity and specificity to identify and quantify viable bacteria in human feces accurately, distinguishing exogenous strains from native microbiota. Through the evaluation of two different formulations of probiotic preparations in healthy individuals, we found that 1.53–6.90% of ZS-8 cells remained viable after the gastrointestinal tract. MLSC significantly improved the gastrointestinal tolerance of ZS-8 compared to powder formulations both *in vitro* and *in vivo*. After ZS-8 administration, the gut microbiota diversity and total viable counts of *Bifidobacterium* and *Lactobacillus* remained stable, and some indigenous *Bifidobacterium* and *Lactobacillus* species increased. These results confirm that PMAxx-qPCR is effective for studying probiotic survival and colonization in the human gut and affirm ZS-8's safety for healthy individuals.

## Data Availability

The sequencing data have been deposited in the National Genomics Data Center (NGDC) under project number PRJCA022875. All data relevant to the study are included in this article or provided as supplemental material.

## SUPPLEMENTAL MATERIAL

Tables S1 to S6 spectrum.02861-24-s0001.docx Additional experimental details.

Table S7 spectrum.02861-24-s0002.csv Raw microbial reads (ASVs).

Table S8 spectrum.02861-24-s0003.csv Rarefied microbial reads (ASVs).

Table S9 spectrum.02861-24-s0004.txt Genome IDs of 553 *B. longum* isolates.

Tables S10 to S13 spectrum.02861-24-s0005.docx Culture method.

Table S14 spectrum.02861-24-s0006.csv Identification results of *Bifidobacterium* species.

Table S15 spectrum.02861-24-s0007.csv Identification results of *Lactobacillus* species.

Table S16 spectrum.02861-24-s0008.docx Individual data of viable and total ZS-8 counts in feces on intervention day 14.

Table S17 spectrum.02861-24-s0009.docx Cell morphology and metabolites of ZS-8.

Supplemental legends spectrum.02861-24-s0010.docx Legends for Tables S1 to S17.

ASM does not own the copyrights to Supplemental Material that may be linked to, or accessed through, an article. The authors have granted ASM a non-exclusive, world-wide license to publish the Supplemental Material files. Please contact the corresponding author directly for reuse.
