# Supplementary material for: Survival and safety evaluation of Bifidobacterium longum subsp. longum ZS-8 in healthy adults, determined using PMAxx-qPCR and amplicon sequencing
Source: Microbiol Spectr. 2025 Sep 22;13(11):e02861-24. doi: 10.1128/spectrum.02861-24 (PMC12584690; doi:10.1128/spectrum.02861-24)
Supplement: Tables S1 to S6 — Additional experimental details. [file spectrum.02861-24-s0001.docx]

| **Species** | **Accession Number** | **Culture Conditions** |
| --- | --- | --- |
| ***Bifidobacterium*** |  |  |
| *B.longum* | ZS-8 | RCA |
| *B.longum* | ATCC15702 | RCA |
| *B.longum* | SZ-7 | RCA |
| B.longum | BB536 | RCA |
| B.longum | HBCICC55020 | RCA |
| B.longum | HBCICC55002 | RCA |
| B.longum | HBCICC 10101 | RCA |
| B.longum | HBCICC55030 | RCA |
| *B.infants* | M-63 | MRS (Modified） |
| *B.latis* | BB12 | MRS (Modified） |
| *B.breve* | M-16V | MRS (Modified） |
| *B.bifidum* | HBCICC52035 | MRS (Modified） |
| *B.adolescentis* | HBCICC54027 | MRS (Modified） |
| *B.animalis* | HBCICC52306 | MRS (Modified） |
| ***Lactobacillus*** |  |  |
| *L. plantarum* | R1012 | MRS |
| *L. acidophilus* | NCFM | MRS |
| *L. reuteri* | LR08 | MRS |
| *L. paracasei* | N115 | MRS |
| *L.gasseri* | HBCICC81001 | MRS |
| *L.casei* | HBCICC 56624 | MRS |
| *L.rhamnosus* | HBCICC51004 | MRS |
| **Non-lactic acid bacteria** |  |  |
| *E. coli* | K12 | LB |
| *S. faecalis* | ATCC 29212 | BHI |
| *S. enterica* | ATCC 14028 | LB |
| *Akkermansia municiphila* | HBCICC59008 | BHI |
| *Enterococcus faecium* | HBCICC82102 | LB |
| *Escherichia coli* | HBCICC21002 | LB |
| *Clostridium sporogenes* | HBCICC85001 | RCM |
| *Enterobacter aerogenes* | HBCICC82017 | LB |
| *Enterococcus faecalis* | HBCICC82929 | LB |
| *Bacillus licheniformis* | HBCICC80302 | TSA |
| *Bacillus amyloliquefaciens* | HBCICC82031 | TSA |
| *Bacillus cereus* | HBCICC 80001 | TSA |
| *Bacillus coagulans* | HBCICC 80172 | TSA |
| *Faecalibacterium prausnitzii* | HBCICC 59006 | YCFA |
| *Clostridium butyricum* | HBCICC 59101 | RCM |
| *Clostridium tyrobutyricum* | HBCICC 59102 | RCM |
| *Bacteroides fragilis* | HBCICC83001 | BHI |
| *Bacteroides vulgatus* | HBCICC 83005 | BHI |
| **Healthy volunteers** |  |  |
| Healthy volunteers 101 | / | / |
| Healthy volunteers 102 | / | / |
| Healthy volunteers 201 | / | / |
| Healthy volunteers 202 | / | / |
| Healthy volunteers 302 | / | / |
| Healthy volunteers 303 | / | / |
| Healthy volunteers 401 | / | / |
| Healthy volunteers 501 | / | / |

Table S1. Bacterial strains and human feces used for primer validation via qPCR

| Strain | Ct | | |
| --- | --- | --- | --- |
| *B.longum* ZS-8 | 13.55 | 13.61 | 13.32 |
| *B.longum* ATCC15702 | 35.09 | 34.58 | 35.20 |
| *B.longum* SZ-7 | 35.99 | 37.81 | 37.24 |
| *B.longum* BB536 | 36.56 | 34.45 | 35.17 |
| *B.longum* HBCICC55020 | 36.60 | 35.71 | 37.12 |
| *B.longum* HBCICC55002 | N/A | 36.50 | 37.01 |
| *B.longum* HBCICC10101 | 37.45 | 35.01 | 37.41 |
| *B.longum* HBCICC55030 | N/A | N/A | N/A |
| *B.infants* M-63 | N/A | N/A | N/A |
| *B.latis* BB12 | 36.25 | N/A | 34.81 |
| *B.breve* M-16V | N/A | N/A | N/A |
| *B.bifidum* SZ-7 | N/A | N/A | N/A |
| *B.adolescentis* HBCICC54027 | 36.12 | 37.81 | 38.01 |
| *B.animalis* HBCICC52306 | 36.24 | 36.21 | N/A |
| *L. plantarum*R1012 | 36.22 | 37.54 | N/A |
| *L. Acidophilus* NCFM | N/A | N/A | N/A |
| *L. Reuteri* LR08 | N/A | N/A | N/A |
| *L. paracasei*N115 | 34.45 | 33.89 | 37.54 |
| *L.gasseri* HBCICC81001 | N/A | N/A | 37.21 |
| *L.casei* HBCICC 56624 | N/A | N/A | N/A |
| *L.rhamnosus* HBCICC51004 | N/A | N/A | 37.98 |
| *E. coli*K12 | 36.28 | N/A | 36.78 |
| *S. faecalis*ATCC 29212 | 34.58 | N/A | N/A |
| *S. enterica*ATCC 14028 | 38.01 | N/A | N/A |
| *Akkermansia municiphila* HBCICC59008 | 36.89 | N/A | N/A |
| *Enterococcus faecium* HBCICC82102 | N/A | N/A | N/A |
| *Escherichia coli* HBCICC21002 | 36.54 | 35.77 | 36.98 |
| *Clostridium sporogenes* HBCICC85001 | 38.65 | 36.45 | 37.11 |
| *Enterobacter aerogenes* HBCICC82017 | N/A | N/A | 36.20 |
| *Enterococcus faecalis* HBCICC82929 | 37.58 | N/A | N/A |
| *Bacillus licheniformis* HBCICC80302 | 35.21 | N/A | N/A |
| *Bacillus amyloliquefaciens* HBCICC82031 | 36.24 | 37.25 | 38.24 |
| *Bacillus cereus* HBCICC80001 | 36.52 | 36.99 | N/A |
| *Bacillus coagulans* HBCICC 80172 | N/A | 37.99 | 38.02 |
| *Faecalibacterium prausnitzii* HBCICC 59006 | 35.90 | 36.21 | 36.21 |
| *Clostridium butyricum* HBCICC 59101 | N/A | N/A | N/A |
| *Clostridium tyrobutyricum* HBCICC 59102 | 36.44 | 37.66 | 35.14 |
| *Bacteroides fragilis* HBCICC83001 | N/A | N/A | N/A |
| *Bacteroides vulgatus* HBCICC 83005 | N/A | N/A | N/A |
| Healthy volunteer 101 (native) | N/A | N/A | N/A |
| Healthy volunteer 101 (spiked-in ZS-8) | 26.32 | 26.32 | 27.00 |
| Healthy volunteer 102 (native) | N/A | N/A | N/A |
| Healthy volunteer 102 (spiked-in ZS-8) | 27.98 | 28.01 | 28.11 |
| Healthy volunteer 201 (native) | N/A | N/A | N/A |
| Healthy volunteer 201 (spiked-in ZS-8) | 25.11 | 25.04 | 25.69 |
| Healthy volunteer 202 (native) | N/A | N/A | N/A |
| Healthy volunteer 202(spiked-in ZS-8) | 25.33 | 25.86 | 25.41 |
| Healthy volunteer 302 (native) | N/A | N/A | N/A |
| Healthy volunteer 302(spiked-in ZS-8) | 26.58 | 26.43 | 26.34 |
| Healthy volunteer 303 (native) | N/A | N/A | N/A |
| Healthy volunteer 303 (spiked-in ZS-8) | 25.77 | 25.83 | 26.12 |
| Healthy volunteer 401 (native) | N/A | N/A | N/A |
| Healthy volunteer 401 (spiked-in ZS-8) | 24.21 | 24.89 | 24.53 |
| Healthy volunteer 501 (native) | N/A | N/A | N/A |
| Healthy volunteer 501 (spiked-in ZS-8) | 25.45 | 25.16 | 25.63 |
| Negative control | N/A | 36.88 | 35.84 |

Table S2. Ct values of the 39 bacteria and 8 healthy volunteers using strain-specific qPCR assays for ZS-8

| Ct （host 1） | | Ct （host 2） | | Ct （host 3） | | Ct （Negative control） | |
| --- | --- | --- | --- | --- | --- | --- | --- |
| 37.10 | N/A | N/A | N/A | N/A | N/A | 36.89 | N/A |
| N/A | 36.09 | N/A | N/A | N/A | N/A | N/A | N/A |
| 35.66 | N/A | N/A | N/A | N/A | N/A | N/A | N/A |
| 36.56 | N/A | N/A | N/A | N/A | N/A | 36.71 | 38.05 |

Table S3. Ct values of ZS-8 in native fecal samples

| lg（CFU) | Ct (host 1) | | Ct (host 2) | | Ct (host 3) | |
| --- | --- | --- | --- | --- | --- | --- |
| 9.81 | 13.22 | 13.33 | 13.06 | 12.88 | 12.98 | 13.57 |
| 8.81 | 15.96 | 15.69 | 14.99 | 15.79 | 15.86 | 15.69 |
| 7.81 | 19.35 | 19.03 | 19.23 | 20.01 | 19.02 | 18.80 |
| 6.81 | 22.88 | 22.58 | 23.01 | 23.01 | 22.99 | 22.42 |
| 5.81 | 26.34 | 26.53 | 26.47 | 26.00 | 26.07 | 25.38 |
| 4.81 | 28.88 | 28.40 | 28.99 | 28.04 | 28.79 | 28.06 |
| 3.81 | 30.99 | 30.89 | 30.84 | 30.04 | 30.50 | 30.79 |

Table S4. Correlation between the number of viable ZS-8 added to fecal samples and the number determined by PMAxx-qPCR

| Strain | Group | Ct (host 1) | | | Ct (host 2) | | | Ct (host3) | | |
| --- | --- | --- | --- | --- | --- | --- | --- | --- | --- | --- |
| viable ZS-8 | without PMAxx | 11.17 | 12.75 | 11.23 | 11.78 | 12.75 | 11.53 | 13.15 | 12.57 | 12.14 |
|  | with PMAxx | 12.02 | 12.48 | 12.26 | 12.63 | 12.02 | 12.14 | 13.39 | 13.51 | 12.96 |
| Dead ZS-8 | without PMAxx | 12.23 | 12.29 | 12.26 | 12.29 | 12.45 | 12.35 | 12.54 | 12.54 | 12.78 |
|  | with PMAxx | 22.33 | 23.70 | 23.00 | 25.77 | 25.16 | 25.71 | 24.31 | 24.70 | 24.37 |

Table S5. Effect of PMAxx on live or dead ZS-8

| Viable count of ZS-8  powder（CFU） | 1# | 2# | 3# | 4# | mean | survival rates（ %） |
| --- | --- | --- | --- | --- | --- | --- |
| 0h | 6.13E+09 | 7.13E+09 | 7.43E+09 | 6.62E+09 | 6.83E+09 | / |
| SGF | <4.92E+03 | <4.93E+03 | <4.98E+03 | <4.99E+03 | <4.95E+03 | <0.000073 |
| SGF+SIF | <4.92E+02 | <4.93E+02 | <4.98E+02 | <4.99E+02 | <4.95E+02 | <0.000007 |
| Viable count of ZS-8  MLSC（CFU） | 1# | 2# | 3# | 4# | mean | survival rates  （%） |
| 0h | 5.23E+08 | 7.66E+08 | 8.32E+08 | 1.32E+09 | 8.61E+08 | / |
| SGF | 1.05E+08 | 7.88E+07 | 1.11E+08 | 1.30E+08 | 1.06E+08 | 12.34 |
| SGF+SIF | 8.96E+06 | 1.52E+07 | 9.97E+06 | 2.27E+07 | 1.42E+07 | 1.65 |

Table S6. The survival rates of ZS-8 after 2 h in SGF or 6 h in SGF+SIF *in vitro*
