# Supplementary material for: Survival and safety evaluation of Bifidobacterium longum subsp. longum ZS-8 in healthy adults, determined using PMAxx-qPCR and amplicon sequencing
Source: Microbiol Spectr. 2025 Sep 22;13(11):e02861-24. doi: 10.1128/spectrum.02861-24 (PMC12584690; doi:10.1128/spectrum.02861-24)
Supplement: Tables S10 to S13 — Culture method. [file spectrum.02861-24-s0005.docx]

| Group | 0d | 7d | 14d | 21d | 28d | 42d |
| --- | --- | --- | --- | --- | --- | --- |
| 1 | 8.48±0.36 | 9.12±0.78* | 9.49±0.57** | 8.75±0.48 | 8.72±0.32 | 8.61±0.55 |
| 2 | 8.57±1.20 | 9.55±0.56 | 10.05±0.43* | 8.74±0.48 | 8.66±0.59 | 8.27±0.52 |
| 3 | 8.89±0.66 | 8.97±0.97 | 9.67±0.49* | 9.35±0.36 | 9.09±0.46 | 9.12±0.75 |
| 4 | 8.97±0.62 | 9.03±0.76 | 9.62±0.39* | 9.07±0.59 | 9.03±0.68 | 9.00±0.91 |
| 5 | 8.74±1.40 | 8.92±1.52 | 9.45±1.27* | 8.89±0.97 | 8.82±1.02 | 8.62±1.30 |

Table S10. Counts of*Bifidobacterium* spp. (log10 CFU (g wet weight feces)^−1^) in feces of volunteers across five groups after 14 days zs-8 ingestion by plate-counting

| Group | Volunteer | 0d | 7d | 14d | 21d | 28d | 42d |
| --- | --- | --- | --- | --- | --- | --- | --- |
| 1 | 101 | 8.42 | 8.97 | 8.51 | 8.74 | 9.02 | 8.60 |
|  | 102 | 7.97 | 8.24 | 10.11 | 8.43 | 8.23 | 8.83 |
|  | 103 | 8.23 | 9.82 | 9.88 | 9.28 | 9.25 | 9.47 |
|  | 104 | 8.59 | 9.46 | 9.47 | 8.97 | 8.68 | 8.02 |
|  | 105 | 8.87 | 8.71 | 10.00 | 8.97 | 8.65 | 7.90 |
|  | 106 | 8.14 | 8.70 | 9.11 | 8.72 | 8.63 | 8.80 |
|  | 107 | 8.59 | 8.50 | 9.86 | 9.13 | 8.45 | 8.18 |
|  | 108 | 9.02 | 10.56 | 8.98 | 7.77 | 8.81 | 9.11 |
| 2 | 401 | 6.41 | 9.56 | 10.45 | 8.96 | 8.07 | 7.38 |
|  | 402 | 8.23 | 9.11 | 9.42 | 7.84 | 8.17 | 8.37 |
|  | 403 | 9.07 | 10.04 | 10.5 | 8.73 | 9.38 | 8.09 |
|  | 404 | 8.48 | 8.67 | 9.7 | 8.86 | 9.36 | 8.84 |
|  | 405 | 9.78 | 9.92 | 10.01 | 9.24 | 8.72 | 8.72 |
|  | 407 | 9.43 | 10.01 | 10.23 | 8.82 | 8.26 | 8.24 |
| 3 | 201 | 7.50 | 7.06 | 10.05 | 9.38 | 8.69 | 8.40 |
|  | 202 | 9.16 | 10.02 | 8.70 | 9.08 | 9.33 | 9.50 |
|  | 203 | 9.57 | 9.46 | 9.88 | 9.13 | 8.79 | 9.81 |
|  | 204 | 8.61 | 8.39 | 9.79 | 9.01 | 9.70 | 8.88 |
|  | 205 | 8.69 | 8.48 | 9.95 | 9.18 | 8.94 | 9.04 |
|  | 206 | 9.33 | 9.84 | 9.81 | 9.78 | 9.54 | 9.93 |
|  | 207 | 9.41 | 9.46 | 10.05 | 10.01 | 9.33 | 9.60 |
|  | 208 | 8.81 | 9.02 | 9.10 | 9.21 | 8.36 | 7.76 |
| 4 | 302 | 8.04 | 8.54 | 9.97 | 8.76 | 8.29 | 9.25 |
|  | 303 | 9.02 | 9.49 | 9.35 | 9.12 | 9.04 | 9.34 |
|  | 304 | 8.21 | 7.70 | 9.59 | 8.03 | 8.19 | 7.16 |
|  | 305 | 9.48 | 9.92 | 10.18 | 9.47 | 8.80 | 8.32 |
|  | 306 | 9.91 | 9.53 | 9.80 | 10.01 | 10.21 | 9.74 |
|  | 307 | 8.79 | 8.94 | 9.67 | 9.09 | 8.90 | 8.81 |
|  | 308 | 8.99 | 8.46 | 9.51 | 9.35 | 9.05 | 9.42 |
| 5 | 310 | 9.30 | 9.67 | 8.89 | 8.73 | 9.73 | 9.99 |
|  | 501 | 7.6 | 8.71 | 10.03 | 8.42 | 7.39 | 8.16 |
|  | 502 | 9.77 | 9.83 | 9.97 | 9.61 | 9.91 | 9.71 |
|  | 504 | 9.65 | 9.34 | 9.94 | 9.65 | 8.86 | 10.23 |
|  | 505 | 8.69 | 9.59 | 9.22 | 8.62 | 8.2 | 7.99 |
|  | 506 | 6.14 | 5.69 | 6.87 | 7.01 | 7.88 | 6.3 |
|  | 507 | 9.61 | 10.29 | 10.21 | 9.67 | 9.74 | 9.25 |
|  | 508 | 9.75 | 8.94 | 10.08 | 9.24 | 9.75 | 8.71 |

Table S11. Individual counts of *Bifidobacterium* spp. (log10 CFU (g wet weight feces)^−1^) in feces of volunteers across five groups after 14 days of zs-8 ingestion by culture methods

| Group | 0d | 7d | 14d | 21d | 28d | 42d |
| --- | --- | --- | --- | --- | --- | --- |
| 1 | 6.44±1.06 | 8.96±0.35**** | 8.30±0.74* | 8.68±0.37**** | 8.54±0.55*** | 8.34±0.70*** |
| 2 | 5.75±1.85 | 8.74±1.03** | 9.02±1.57** | 8.44±0.98** | 7.72±1.48* | 7.73±0.88* |
| 3 | 8.00±1.42 | 9.09±0.92* | 8.86±0.89 | 9.44±0.82* | 8.92±0.48 | 9.07±0.56* |
| 4 | 6.16±1.80 | 8.45±1.29** | 8.87±0.87*** | 8.42±1.58** | 8.65±1.37** | 8.14±1.48 |
| 5 | 6.99±1.57 | 8.84±1.01** | 8.72±1.00* | 8.92±1.02* | 7.99±1.82 | 7.87±1.81 |

Table S12. Counts of *Lactobacillus* spp. (log10 CFU (g wet weight feces)^−1^) in feces of volunteers across five groups after 14 days zs-8 ingestion by culture methods

| Group | Volunteer | 0d | 7d | 14d | 21d | 28d | 42d |
| --- | --- | --- | --- | --- | --- | --- | --- |
| 1 | 101 | 5.89 | 8.67 | 7.65 | 9.11 | 8.90 | 8.40 |
|  | 102 | 5.50 | 8.77 | 8.08 | 8.34 | 8.90 | 7.09 |
|  | 103 | 7.36 | 9.51 | 7.62 | 8.52 | 8.85 | 9.32 |
|  | 105 | 7.74 | 8.90 | 7.56 | 8.75 | 8.35 | 7.91 |
|  | 106 | 6.28 | 9.08 | 9.13 | 8.98 | 7.41 | 8.58 |
|  | 107 | 7.34 | 9.28 | 9.16 | 8.97 | 8.94 | 8.30 |
|  | 108 | 4.98 | 8.50 | 8.92 | 8.11 | 8.46 | 8.77 |
| 2 | 401 | 4.71 | 7.11 | 10.66 | 9.28 | 7.86 | 6.24 |
|  | 402 | 4.70 | 9.00 | 9.49 | 7.88 | 7.16 | 8.15 |
|  | 403 | 4.70 | 9.71 | 9.64 | 8.76 | 9.00 | 7.13 |
|  | 404 | 5.72 | 8.13 | 9.57 | 8.86 | 8.89 | 9.03 |
|  | 405 | 9.75 | 9.49 | 9.24 | 9.22 | 8.07 | 8.18 |
|  | 407 | 5.99 | 9.80 | 8.88 | 8.60 | 8.41 | 7.80 |
|  | 408 | 4.67 | 7.95 | 5.67 | 6.48 | 4.68 | 7.57 |
| 3 | 201 | 6.37 | 7.38 | 7.20 | 8.79 | 8.77 | 8.36 |
|  | 202 | 8.69 | 10.41 | 7.96 | 9.53 | 8.34 | 9.34 |
|  | 203 | 9.10 | 8.98 | 9.01 | 10.08 | 9.73 | 9.65 |
|  | 204 | 8.05 | 8.25 | 8.92 | 10.22 | 9.17 | 8.78 |
|  | 205 | 8.31 | 9.27 | 10.06 | 7.75 | 8.63 | 8.85 |
|  | 206 | 9.22 | 9.48 | 9.23 | 9.63 | 9.21 | 9.80 |
|  | 207 | 8.97 | 9.72 | 9.16 | 10.09 | 9.17 | 9.41 |
|  | 208 | 5.29 | 9.25 | 9.37 | 9.43 | 8.37 | 8.38 |
| 4 | 302 | 4.64 | 8.68 | 8.08 | 9.20 | 8.65 | 9.23 |
|  | 303 | 5.32 | 5.67 | 7.23 | 4.69 | 9.19 | 8.83 |
|  | 304 | 6.07 | 7.77 | 8.59 | 8.75 | 7.90 | 8.68 |
|  | 305 | 8.25 | 9.94 | 10.16 | 9.35 | 8.84 | 8.29 |
|  | 306 | 9.09 | 9.44 | 9.33 | 9.76 | 10.16 | 5.70 |
|  | 307 | 8.02 | 9.04 | 9.41 | 9.15 | 9.24 | 8.76 |
|  | 308 | 4.64 | 8.34 | 9.36 | 8.98 | 8.78 | 8.00 |
|  | 309 | 4.72 | 7.69 | 8.51 | 7.09 | 5.42 | 5.76 |
|  | 310 | 4.69 | 9.47 | 9.21 | 8.86 | 9.63 | 10.03 |
| 5 | 501 | 4.64 | 8.68 | 8.08 | 9.20 | 8.65 | 9.23 |
|  | 502 | 5.32 | 5.67 | 7.23 | 4.69 | 9.19 | 8.83 |
|  | 504 | 6.07 | 7.77 | 8.59 | 8.75 | 7.9 | 8.68 |
|  | 505 | 8.25 | 9.94 | 10.16 | 9.35 | 8.84 | 8.29 |
|  | 506 | 9.09 | 9.44 | 9.33 | 9.76 | 10.16 | 5.7 |
|  | 507 | 8.02 | 9.04 | 9.41 | 9.15 | 9.24 | 8.76 |
|  | 508 | 4.64 | 8.34 | 9.36 | 8.98 | 8.78 | 8.00 |

Table S13. Individual counts of *Lactobacillus* spp. (log10 CFU (g wet weight feces)^−1^)in feces of volunteers across five groups after 14 days of zs-8 ingestion by culture methods
