# Supplementary material for: Survival and safety evaluation of Bifidobacterium longum subsp. longum ZS-8 in healthy adults, determined using PMAxx-qPCR and amplicon sequencing
Source: Microbiol Spectr. 2025 Sep 22;13(11):e02861-24. doi: 10.1128/spectrum.02861-24 (PMC12584690; doi:10.1128/spectrum.02861-24)
Supplement: Table S16 — Individual data of viable and total ZS-8 counts in feces on intervention day 14. [file spectrum.02861-24-s0008.docx]

| Group | Volunteer | Log 10 CFU (live+dead)/g feces | Log 10 CFU (live)/g feces | Viable percent (%) |
| --- | --- | --- | --- | --- |
| 1 | 101 | 7.95 | 6.10 | 1.41% |
|  | 102 | 7.70 | 5.04 | 0.22% |
|  | 103 | 7.56 | 5.84 | 1.91% |
|  | 105 | 7.11 | 5.44 | 2.14% |
|  | 106 | 7.89 | 6.23 | 2.19% |
|  | 107 | 8.99 | 6.02 | 0.11% |
|  | 108 | 8.78 | 7.31 | 3.39% |
| 2 | 401 | 7.43 | 6.04 | 4.07% |
|  | 402 | 9.00 | 7.03 | 1.07% |
|  | 403 | 7.96 | 5.89 | 0.85% |
|  | 404 | 8.86 | 6.26 | 0.25% |
|  | 405 | 8.43 | 5.95 | 0.33% |
|  | 407 | 8.64 | 6.92 | 1.91% |
| 3 | 201 | 7.07 | 6.05 | 9.55% |
|  | 203 | 7.54 | 6.98 | 27.54% |
|  | 204 | 7.36 | 5.65 | 1.95% |
|  | 205 | 6.89 | 5.85 | 9.12% |
|  | 206 | 6.69 | 5.59 | 7.94% |
|  | 207 | 7.84 | 6.05 | 1.62% |
| 4 | 301 | 9.10 | 7.94 | 6.92% |
|  | 302 | 9.24 | 7.86 | 4.17% |
|  | 303 | 9.83 | 8.18 | 2.24% |
|  | 304 | 8.74 | 7.23 | 3.09% |
|  | 305 | 8.38 | 7.34 | 9.12% |
|  | 306 | 8.59 | 7.48 | 7.76% |
|  | 307 | 7.73 | 6.46 | 5.37% |
|  | 308 | 7.81 | 6.41 | 3.98% |
|  | 309 | 8.96 | 8.00 | 10.96% |
| 5 | 501 | 8.80 | 6.69 | 0.78% |
|  | 502 | 8.11 | 6.64 | 3.39% |
|  | 504 | 8.98 | 7.50 | 3.31% |
|  | 505 | 8.20 | 7.45 | 17.78% |
|  | 507 | 8.53 | 6.70 | 1.48% |

Table S16 Individual data of viable and total ZS-8 counts in feces on intervention day 14
