# Supplementary material for: Survival and safety evaluation of Bifidobacterium longum subsp. longum ZS-8 in healthy adults, determined using PMAxx-qPCR and amplicon sequencing
Source: Microbiol Spectr. 2025 Sep 22;13(11):e02861-24. doi: 10.1128/spectrum.02861-24 (PMC12584690; doi:10.1128/spectrum.02861-24)
Supplement: Table S17 — Cell morphology and metabolites of ZS-8. [file spectrum.02861-24-s0009.docx]

| Metabolite Name | Medium Control  (µmol/L) | ZS-8 (µmol/L) | BB536 (µmol/L) |
| --- | --- | --- | --- |
| Lactic acid | 2987.525 | 22025.080 | 18908.467 |
| Acetic acid | 27098.403 | 50670.164 | 49290.694 |
| Phenyllactic acid | 1.435 | 163.045 | 159.526 |
| 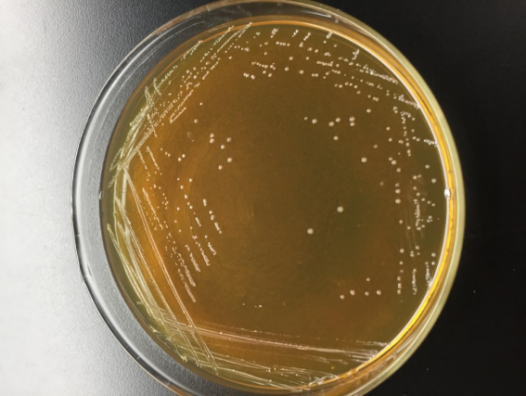**Colony Morphology** | | 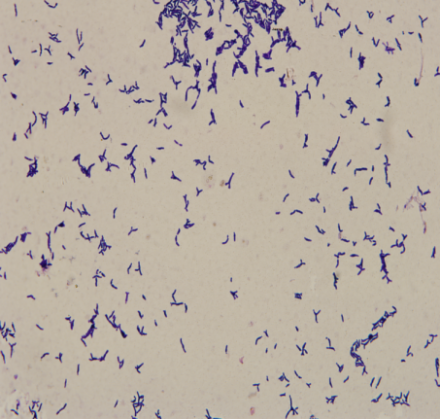Cellular morphology | |

Table S17 **Cell morphology and some metabolites of ZS-8**
