# Supplementary material for: Survival and safety evaluation of Bifidobacterium longum subsp. longum ZS-8 in healthy adults, determined using PMAxx-qPCR and amplicon sequencing
Source: Microbiol Spectr. 2025 Sep 22;13(11):e02861-24. doi: 10.1128/spectrum.02861-24 (PMC12584690; doi:10.1128/spectrum.02861-24)
Supplement: Supplemental legends — Legends for Tables S1 to S17. [file spectrum.02861-24-s0010.docx]

**Table S1**. Bacterial strains and human feces used for primer validation via qPCR

**Table S2.** Ct values of the 39 bacteria using strain-specific qPCR assays for ZS-8

**Table S3.** Ct values of ZS-8 in native fecal samples

**Table S4.** Correlation between the number of viable ZS-8 added to fecal samples and the number determined by PMAxx-qPCR

**Table S5.** Effect of PMAxx on live or dead ZS-8

**Table S6**. The survival rates of ZS-8 after 2 h in SGF or 6 h in SGF+SIF *in vitro*

**Table S7.** Raw microbial reads (ASVs)

**Table S8.** Rarefied microbial reads (ASVs)

**Table S9**. Genome ID of 553 *B.longum*

**Table S10.** Counts of*Bifidobacterium* spp. (log10 CFU (g wet weight feces)^−1^) in feces of volunteers across five groups after 14 days ZS-8 ingestion by plate-counting

**Table S11.** Individual counts of *Bifidobacterium* spp. (log10 CFU (g wet weight feces)^−1^) in feces of volunteers across five groups after 14 days of ZS-8 ingestion by culture methods

**Table S12.** Counts of *Lactobacillus* spp. (log10 CFU (g wet weight feces)^−1^) in feces of volunteers across five groups after 14 days ZS-8 ingestion by culture methods

**Table S13**. Individual counts of *Lactobacillus* spp. (log10 CFU (g wet weight feces)^−1^) in feces of volunteers across five groups after 14 days of ZS-8 ingestion by culture methods

**Table S14**. Identification results of *Bifidobacterium* species

**Table S15**. Identification results of *Lactobacillus* species

**Table S16.** Individual data of viable and total ZS-8 counts in feces on intervention day 14

**Table S17.** Cell morphology and some metabolites of ZS-8
